# Supplementary material for: Filtered mud improves sugarcane growth and modifies the functional abundance and structure of soil microbial populations
Source: PeerJ. 2022 Jan 13;10:e12753. doi: 10.7717/peerj.12753 (PMC8771795; doi:10.7717/peerj.12753)
Supplement: Supplemental Information 4 — CK (control), FM: Filtered mud, FM1 (FM:soil at 1:4), FM2 (FM:soil at 2:3), FM3 (FM:soil at 3:2). [file peerj-10-12753-s004.docx]

**Filtered mud improves sugarcane growth and modifies the functional abundance and structure of soil microbial populations**

Ahmad Yusuf Abubakar^1,2,3^, Muhammed Mustapha Ibrahim^4,5^, Caifang Zhang^1,2^, Muhammad Tayyab^1,2^, Nyumah Fallah^1,2^, Ziqi Yang^1,2^, Ziqin Pang^1,2^, Hua Zhang^1,2,^*

^1^ Key Laboratory of Sugarcane Biology and Genetic Breeding, Ministry of Agriculture, Fujian Agriculture and Forestry University, Fuzhou, China

^2^ College of Agriculture, Fujian Agriculture and Forestry University, Fuzhou, China

^3^ Bioresources Development Centre, Kano; National Biotechnology Development Agency (NABDA) Abuja, Nigeria

^4^ Department of Soil Science, University of Agriculture, Makurdi, Nigeria

^5^ Key Research Laboratory of Soil Ecosystem Health and Regulation in Fujian Provincial University, College of Resources and Environment, Fujian Agriculture and Forestry University, Fuzhou, Fujian Province, China.

*Corresponding author

Email: zhanghua4553@sina.com (H.Zhang)

**Supplementary material**

Fig S1. Relative abundance of soil bacterial community at genus level. CK (control), FM: Filtered mud, FM1 (FM:soil at 1:4), FM2 (FM:soil at 2:3), FM3 (FM:soil at 3:2).

Fig S2. Relative abundance of soil fungal community at genus level. CK (control), FM: Filtered mud, FM1 (FM:soil at 1:4), FM2 (FM:soil at 2:3), FM3 (FM:soil at 3:2).
